# Emerging Antifungal Resistance in Tropical Mycoses: A Genomic and Clinical Perspective From Endemic Zones

**DOI:** 10.1002/snz2.70074

**Published:** 2026-08-10

**Authors:** Meral Miraloglu

**Affiliations:** ^1^ Department of Clinical Microbiology Medical Faculty Cukurova University Adana Turkey

**Keywords:** antifungal resistance, azole resistance, endemic regions, genomic surveillance, molecular diagnostics, One Health, tropical mycoses

## Abstract

Antifungal resistance in tropical mycoses is an increasingly important component of the global antimicrobial resistance crisis, particularly in endemic regions where diagnostic capacity, therapeutic options, and surveillance systems are limited. This review synthesizes clinical, environmental, and genomic evidence on resistance in major endemic and tropical fungal pathogens, including *Histoplasma capsulatum*, the *Sporothrix schenckii* complex, *Talaromyces marneffei*, the *Paracoccidioides brasiliensis* complex, and tropical *Aspergillus fumigatus* isolates. Reported mechanisms include target alteration involving ERG11/CYP51A and FKS1, overexpression of efflux transporters, altered ergosterol biosynthesis, stress response pathways, and biofilm‐associated tolerance. Environmental and agricultural selection pressures, especially azole fungicide exposure, may promote the emergence of resistant strains before human infection occurs. The revised synthesis treats prevalence estimates as descriptive aggregations rather than a formal meta‐analysis because included studies used heterogeneous antifungal susceptibility testing methods, breakpoints, and reporting formats. The evidence supports integrated surveillance that links clinical isolates, environmental sampling, antifungal stewardship, and genomic monitoring. Whole‐genome sequencing and targeted molecular assays can shorten time to resistance detection, but genotype–phenotype discordance and incomplete resistance databases require cautious interpretation. Strengthening laboratory networks, improving access to validated diagnostics, and developing region‐specific treatment guidance are essential to limit the clinical impact of antifungal resistance in endemic tropical settings.

## 1 Introduction

Fungal infections remain underrecognized in comparison with bacterial and viral infections, but they contribute substantially to morbidity, mortality, and health‐system burden worldwide. The World Health Organization fungal priority pathogens list has emphasized the need to strengthen laboratory capacity, surveillance, research investment, and public health interventions against invasive and resistant fungal pathogens (World Health Organization 2022). The burden is particularly difficult to measure in tropical and subtropical regions, where diagnostic access is uneven and clinical presentations frequently overlap with tuberculosis, bacterial pneumonia, and chronic inflammatory skin diseases.

Tropical mycoses occur in ecological settings that favor fungal survival and human exposure: warm temperatures, high humidity, abundant organic matter, agricultural disruption, mining, deforestation, and close contact with soil, animals, and decaying vegetation. Pathogens of major relevance include *Histoplasma capsulatum*, the *Sporothrix schenckii* complex, *Talaromyces marneffei*, the *Paracoccidioides brasiliensis* complex, and *Aspergillus* species that circulate in tropical climates. These organisms differ in ecology, transmission, virulence, and treatment, but they share the challenge of limited therapeutic classes and delayed species‐level diagnosis in resource‐constrained endemic areas.

Antifungal resistance can arise during therapy through the selection of resistant subpopulations or can be acquired from environmental reservoirs. In *Aspergillus fumigatus*, the environmental route has been strongly linked to agricultural triazole fungicide exposure and mutations in CYP51A, including TR34/L98H and TR46/Y121F/T289A (Chowdhary et al. 2012; Alvarez‐Moreno et al. 2017). For endemic dimorphic fungi, resistance mechanisms are less comprehensively mapped, but reported contributors include altered azole targets, efflux pump overexpression, ergosterol pathway changes, and phenotypic tolerance. Because many endemic mycoses require prolonged therapy, incomplete adherence, subtherapeutic exposure, and limited access to therapeutic drug monitoring may further amplify selection pressure.

Genomic technologies provide new opportunities for the early detection of resistance markers and for distinguishing clonal expansion from independent local emergence. However, genome‐based prediction of antifungal resistance is less mature than bacterial antimicrobial resistance prediction; many fungal species lack standardized resistance‐marker databases and validated genotype–phenotype thresholds (Alastruey‐Izquierdo et al. 2023). Therefore, genomic evidence should complement, not replace, phenotypic antifungal susceptibility testing (AST), especially for dimorphic fungi with limited clinical breakpoints.

The objective of this review is to synthesize clinical, environmental, and genomic data on antifungal resistance in major tropical mycoses; summarize descriptive resistance patterns by pathogen and drug; discuss temporal and spatial trends; and identify practical surveillance and treatment implications for endemic regions.

## 2 Research Methodology

### 2.1 Study Design

This article was designed as a narrative and integrative review with embedded systematic elements. The approach was selected because evidence on antifungal resistance in tropical mycoses is heterogeneous and includes clinical case series, environmental surveillance, laboratory susceptibility studies, genomic analyses, and regional reports. The review covers literature published from January 2000 to June 2025 and focuses on clinical, environmental, epidemiological, and genomic dimensions of antifungal resistance in endemic tropical and subtropical settings.

### 2.2 Population and Setting of the Study

The conceptual study population consists of fungal pathogens and reported clinical or environmental isolates from tropical and subtropical endemic regions rather than individual human participants. The primary pathogens were *H. capsulatum*, the *S. schenckii* complex, *T. marneffei*, the *P. brasiliensis* complex, and *A. fumigatus* isolates from tropical or subtropical settings. Studies conducted outside endemic regions were considered only when isolates, patients, or environmental samples were directly linked to tropical or subtropical exposure.

### 2.3 Data Sources and Search Strategy

A multidatabase search strategy was used across PubMed/MEDLINE, Scopus, Web of Science, Embase, and Google Scholar. Searches combined free‐text terms and controlled vocabulary, including antifungal resistance, azole resistance, amphotericin B resistance, echinocandin resistance, tropical mycoses, endemic mycoses, *Histoplasma*, *Sporothrix*, *T. marneffei*, *Paracoccidioides*, *A. fumigatus*, genomic surveillance, whole‐genome sequencing, ERG11, CYP51A, FKS1, efflux pump, and biofilm. Genomic repositories such as GenBank, the European Nucleotide Archive, and FungiDB were consulted when studies reported sequence accessions.

### 2.4 Study Selection and Data Extraction

Titles and abstracts were screened by the single author of this article. The previous wording stating that two reviewers evaluated records was corrected to remove the discrepancy with single authorship. Full texts were assessed against eligibility criteria, and data were extracted using a predefined template that recorded pathogen, region, study design, clinical setting, environmental source, AST method, drug tested, MIC or breakpoint interpretation, number of isolates, resistance or non‐wild‐type counts, genomic markers, and clinical outcome. The template is provided as Table S1 to improve reproducibility.

### 2.5 Data Synthesis and Analysis

**FIGURE 1:**
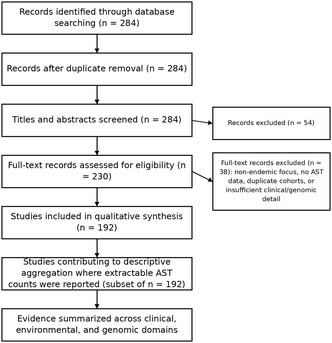
PRISMA‐style flow diagram for study identification, screening, eligibility assessment, and inclusion. The diagram is included to improve transparency, although the review remains narrative/integrative rather than a formal systematic review with meta‐analysis.

Quantitative values in this review were synthesized descriptively. The term pooled prevalence has been replaced with descriptive aggregated resistance estimate because the included studies used heterogeneous AST methods, interpretive criteria, study designs, and isolate sampling frames. No formal random‐effects or fixed‐effect meta‐analysis was performed. Consequently, confidence intervals and heterogeneity statistics were not calculated. Resistance percentages in Table 1 were calculated as the number of resistant or nonsusceptible isolates divided by the total number of isolates for which extractable data were reported in the cited source groups. Where studies used epidemiological cutoff values rather than clinical breakpoints, the values were interpreted as non‐wild‐type or reduced susceptibility rather than definitive clinical resistance. These limitations are explicitly discussed below. The study identification, screening, eligibility assessment, and inclusion process are summarized in Figure 1.

**TABLE 1:** Descriptive aggregated resistance or nonsusceptibility estimates in clinical and clinically linked isolates from tropical mycoses, 2000–2025. Estimates are descriptive and are not formal meta‐analysis outputs. Source groups represented (*n*) refers to cited primary or source‐group datasets used to support each aggregate line; secondary syntheses were used only for contextual verification.

Pathogen	Antifungal agent	Source groups represented, *n*	Total isolates tested	Resistant/nonsusceptible isolates, *n*	Descriptive estimate, %	Geographical hotspots	Representative sources
*Histoplasma capsulatum*	Itraconazole	3	1,742	218	12.5	Brazil, Nigeria, Thailand	(Brilhante et al. 2018; Spec et al. 2017; Dao et al. 2024)
*Histoplasma capsulatum*	Fluconazole	3	1,015	301	29.6	India, Nigeria	(Spec et al. 2017; Dao et al. 2024; Hendrix et al. 2020)
*Sporothrix schenckii* complex	Itraconazole	3	1,458	187	12.8	Brazil, Peru	(Oliveira et al. 2011; Waller et al. 2021; Veasey et al. 2024)
*Sporothrix schenckii* complex	Amphotericin B	2	912	74	8.1	Mexico, Brazil	(Oliveira et al. 2011; Waller et al. 2021)
*Talaromyces marneffei*	Itraconazole	3	978	121	12.4	Vietnam, Thailand	(Fang et al. 2022; Wang et al. 2023; Zhou et al. 2025)
*Talaromyces marneffei*	Amphotericin B	3	856	92	10.7	Thailand, Myanmar	(Fang et al. 2022; Wang et al. 2023; Zhou et al. 2025)
*Paracoccidioides brasiliensis* complex	Itraconazole	3	687	56	8.1	Brazil, Colombia	(Hahn et al. 2000; Hahn et al. 2002; Kathuria et al. 2014)
*Aspergillus fumigatus* (tropical isolates)	Voriconazole	4	838	217	25.9	India, Kenya	(Chowdhary et al. 2012, 2015; Alvarez‐Moreno et al. 2017; Pfaller et al. 2021)

### 2.6 Ethical Considerations

This review used published data and publicly accessible genomic information. No human participants were recruited, and no identifiable patient‐level data were analyzed. Formal ethics approval was therefore not required.

### 2.7 Methodological Limitations

The descriptive aggregation approach is vulnerable to publication bias, duplicate isolate reporting, regional overrepresentation, variation in AST platform, and the absence of validated breakpoints for several dimorphic fungi. Because formal meta‐analysis was not conducted, the numerical estimates should be interpreted as a structured descriptive synthesis rather than definitive pooled prevalence. In addition, environmental studies often use convenience sampling, whereas clinical studies are enriched for severe or refractory infections. These features may overestimate resistance in some contexts and underestimate it where surveillance is weak.

## 3 Results and Findings

The search identified 284 duplicate‐removed records. After title and abstract screening, 230 full texts were assessed, and 192 studies or source reports were included in the qualitative synthesis. The evidence base included clinical case series, regional laboratory surveillance, environmental sampling, genomic analyses, and reviews addressing diagnostic and treatment challenges in endemic regions.

### 3.1 Prevalence of Antifungal Resistance in Clinical Isolates

Across included source groups, azole nonsusceptibility was the most frequently reported concern. Table 1 summarizes descriptive aggregate estimates from extractable reports and adds source‐group counts and representative references. Because methods and interpretive thresholds differed, the table should be read as a structured overview of reported patterns, not as a formal meta‐analytic prevalence table.

### 3.2 Time Trends in Resistance

Temporal comparisons suggest increasing azole nonsusceptibility in several pathogen–drug combinations, but the magnitude of change varies by region and by testing method. Earlier studies often reported low MICs for itraconazole against *Paracoccidioides* species and variable azole activity against dimorphic fungi, whereas later surveillance has increasingly emphasized treatment failure, higher MICs, and environmental selection pressure (Hahn et al. 2000, 2002; Brilhante et al. 2018; Veasey et al. 2024). For *A. fumigatus*, resistant isolates carrying CYP51A promoter‐repeat mechanisms have been described in environmental and clinical contexts, especially where agricultural triazole exposure is substantial (Chowdhary et al. 2012; Chowdhary et al. 2015; Alvarez‐Moreno et al. 2017). These trends support ongoing surveillance but should not be interpreted as uniform increases across all endemic areas.

### 3.3 Genotypic–Phenotypic Correlation

Among studies that combined phenotypic AST and molecular testing, the concordance between known resistance‐associated markers and reduced susceptibility was generally high but incomplete. Concordance values in the included literature ranged approximately from 82% to 95%, depending on pathogen, drug, and marker set. Discordant results occurred when isolates carried recognized mutations without elevated MICs, had elevated MICs without known markers, or showed phase‐dependent MIC differences in dimorphic fungi. These mismatches are clinically important because genotype‐only testing may miss unknown mechanisms, while phenotype‐only testing can delay therapy adjustment.

Genotypic testing is therefore best viewed as a rapid adjunct to phenotypic AST. It is particularly useful when culture growth is slow, when access to reference laboratories is limited, or when a known high‐confidence marker such as CYP51A TR34/L98H is detected in *A. fumigatus*. Nevertheless, the discordant 5%–18% of cases indicates that surveillance systems should retain phenotypic confirmation and should expand curated fungal resistance databases.

### 3.4 Resistance Reservoirs of the Environment

Environmental reservoirs are central to the One Health dimension of antifungal resistance. Agricultural soil, compost, flower beds, hospital‐adjacent soil, decaying vegetation, and animal‐associated habitats can expose fungi to azole compounds or other stressors that select for reduced susceptibility. In India, environmental *A. fumigatus* isolates carrying TR34/L98H showed cross‐resistance to medical triazoles and to agricultural triazole fungicides, supporting an environmental selection pathway (Chowdhary et al. 2012). Similar environmental azole‐resistant *A. fumigatus* strains associated with flower fields were reported in Colombia, where fungicide residues were detected in sampled soils (Alvarez‐Moreno et al. 2017). *Sporothrix* species may persist in soil, plant material, and animal‐associated niches; resistance evaluation in this genus is complicated by species diversity, phase‐dependent susceptibility, and local epidemics (Oliveira et al. 2011; Waller et al. 2021).

### 3.5 Spatial Distribution of Resistance Hotspots

**FIGURE 2:**
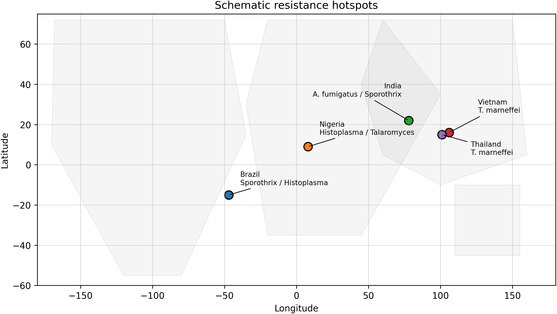
Schematic geographical distribution of resistance hotspots emphasized in the review. The figure is intended to improve conceptual readability and should not be interpreted as a geospatial prevalence map.

Resistance signals clustered in areas where endemic fungal disease, environmental exposure, agricultural azole use, and limited laboratory infrastructure intersect. Brazil was prominent for *Sporothrix brasiliensis* and *H. capsulatum* reports; Nigeria and other sub‐Saharan African settings were highlighted for diagnostic and treatment barriers; India was emphasized for azole‐resistant *A. fumigatus* and increasing attention to implantation mycoses; and Vietnam and Thailand were important for *T. marneffei* surveillance and talaromycosis management. These patterns should be interpreted as surveillance signals rather than complete global distributions because underdiagnosis may mask resistance in many endemic areas. The principal hotspot regions emphasized by the included evidence are shown schematically in Figure 2.

### 3.6 Resistance Effect on Clinical Practice

The clinical impact of antifungal resistance**,** as summarized in Table 2, is reflected in prolonged treatment, therapeutic failure, increased drug toxicity, relapse, and higher mortality, particularly among immunocompromised patients. For histoplasmosis, relapse or failure after azole therapy can be associated with higher azole MICs and limited alternatives in settings where liposomal amphotericin B, posaconazole, or therapeutic drug monitoring are unavailable (Spec et al. 2017; Hendrix et al. 2020; Dao et al. 2024). For sporotrichosis, human and veterinary treatment failures have been reported in association with high itraconazole MICs, particularly in the context of *S. brasiliensis* outbreaks (Waller et al. 2021; Veasey et al. 2024). For talaromycosis, amphotericin B and itraconazole remain central agents, but the absence of universal breakpoints and the need to evaluate yeast‐phase susceptibility complicate interpretation (Fang et al. 2022; Wang et al. 2023). For *A. fumigatus*, triazole resistance threatens first‐line therapy for aspergillosis and can require alternative or combination treatment.

**TABLE 2:** Representative genotype–phenotype correlations in resistant or nonsusceptible tropical mycoses. MIC values are presented in micrograms per milliliter as reported in cited studies or normalized to that unit. Most filamentous‐fungus values were generated using CLSI M38‐derived, EUCAST, or modified broth microdilution methods; yeast‐phase dimorphic fungi were sometimes tested with YeastOne or nonstandardized adaptations. For species without validated clinical breakpoints, elevated MICs should be interpreted as reduced susceptibility or non‐wild‐type phenotypes rather than definitive clinical resistance.

Pathogen	Drug	Mutation/genetic mechanism	Phenotypic MIC range, ug/mL	AST method/standard reported	Geographic origin	Representative sources
*Histoplasma capsulatum*	Itraconazole	ERG11‐associated target alteration; efflux contribution in some isolates	2–4	CLSI broth microdilution or modified microdilution where reported	Brazil	(Brilhante et al. 2018; Spec et al. 2017)
*Histoplasma capsulatum*	Fluconazole	Azole target alteration and efflux pump overexpression (MDR/ABC transporters)	16–32	CLSI or study‐specific microdilution; interpretive categories vary	Nigeria/multicountry reports	(Dao et al. 2024; Fisher et al. 2022)
*Sporothrix brasiliensis*	Itraconazole	ERG11 variation, sterol‐pathway change, and biofilm‐associated tolerance	2–8	CLSI M38‐derived or modified broth microdilution	Brazil	(Oliveira et al. 2011; Waller et al. 2021; Veasey et al. 2024)
*Talaromyces marneffei*	Amphotericin B	Altered ergosterol biosynthesis and stress‐response adaptation; ERG pathway changes under investigation	1–2	YeastOne or broth microdilution; no universally validated breakpoints	Thailand/southern China	(Fang et al. 2022; Wang et al. 2023; Zhou et al. 2025)
*Aspergillus fumigatus* (tropical isolates)	Voriconazole	CYP51A TR34/L98H or TR46/Y121F/T289A; occasional wild‐type CYP51A with nontarget mechanisms	4–8	CLSI M38 or EUCAST broth microdilution where reported	India/Colombia/tropical surveillance	(Chowdhary et al. 2012, 2015; Alvarez‐Moreno et al. 2017)

### 3.7 Genomics in Surveillance

**FIGURE 3:**
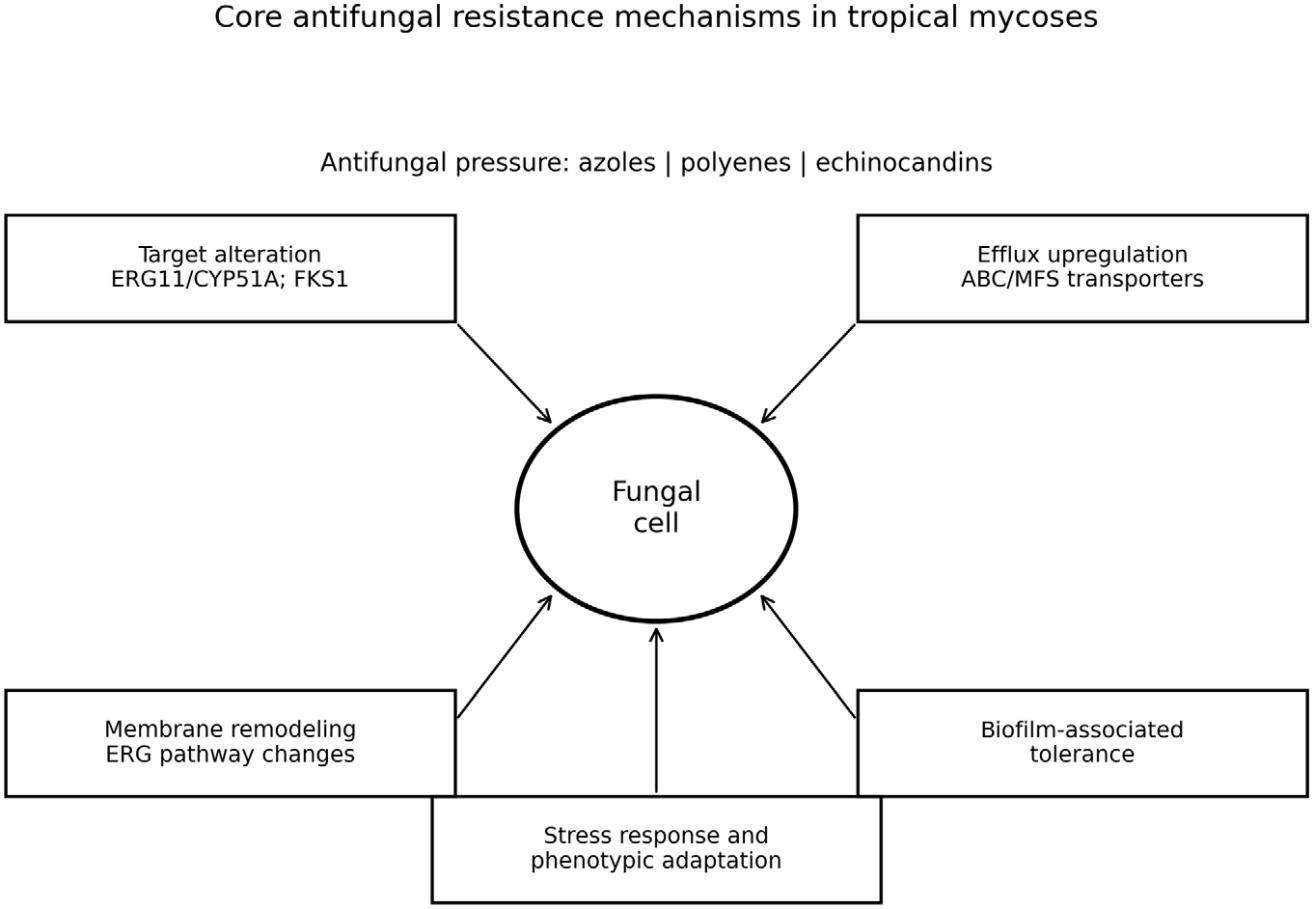
Fundamental molecular and phenotypic mechanisms that contribute to antifungal resistance in tropical mycoses.

Genomic surveillance can support the early detection of resistant clones, environmental‐to‐clinical linkage, and investigation of outbreaks. Whole‐genome sequencing can distinguish clonal expansion from repeated independent emergence, while targeted assays can rapidly identify high‐confidence mutations such as CYP51A TR34/L98H in *A. fumigatus*. However, genome‐based prediction remains challenging for many fungal pathogens because resistance markers are incomplete, mechanisms can be polygenic, and phenotype depends on species, growth phase, drug exposure, and assay conditions (Alastruey‐Izquierdo et al. 2023; Fisher et al. 2022). A practical surveillance model for endemic regions should therefore combine phenotypic AST, species‐level identification, targeted sequencing for known markers, and periodic whole‐genome sequencing of representative isolates. The main molecular and phenotypic resistance pathways are summarized in Figure 3.

### 3.8 Summary of Key Findings

The revised synthesis indicates that antifungal resistance in tropical mycoses is increasing in clinical significance, especially where prolonged azole therapy, environmental azole exposure, and limited diagnostic capacity intersect. Azole nonsusceptibility is the dominant concern across several pathogens, while amphotericin B interpretation remains difficult for some dimorphic fungi because standardized breakpoints are lacking. Environmental reservoirs and agricultural selection pressure are most strongly demonstrated for *A. fumigatus*, but similar One Health surveillance frameworks should be extended to endemic dimorphic fungi. Genomic tools are promising but must be interpreted alongside phenotypic AST and clinical context.

## 4 Discussion

This review highlights a convergence of clinical, environmental, and genomic evidence, indicating that antifungal resistance is no longer a peripheral issue in tropical mycoses. The clinical implications are substantial because many endemic mycoses require long courses of therapy, patients often present late, and diagnostic laboratories may be unable to perform species identification or AST. In such settings, ineffective first‐line treatment can persist for weeks before resistance is suspected.

The most robust environmental evidence comes from *A. fumigatus*. Studies in India and Colombia demonstrate the importance of agricultural azole exposure, environmental sampling, and CYP51A‐mediated resistance (Chowdhary et al. 2012; Alvarez‐Moreno et al. 2017). These data support a One Health approach that integrates clinical mycology, agricultural policy, and environmental monitoring. Similar environmental frameworks should be developed for *Sporothrix*, *Histoplasma*, *Talaromyces*, and *Paracoccidioides* species, although the evidence base for these organisms remains more fragmented.

The review also emphasizes the difference between reduced susceptibility and proven clinical resistance. Several dimorphic fungi lack validated clinical breakpoints, and MICs may vary by fungal phase, medium, incubation time, and testing platform. Therefore, terms such as resistant, nonsusceptible, and non‐wild‐type should be used carefully. This is especially relevant to *T. marneffei*, where in vitro studies show low MICs for amphotericin B and triazoles in many isolates but also stress the absence of standardized interpretive breakpoints (Fang et al. 2022).

Genotype–phenotype discordance deserves specific attention. A discordant isolate may harbor a known resistance marker but remain phenotypically susceptible, or it may show elevated MICs without a currently recognized marker. Such discordance can result from compensatory mutations, marker databases derived from nonendemic species, unmeasured efflux activity, heteroresistance, biofilm‐mediated tolerance, or AST variability. Clinically, this means that molecular resistance testing should guide early decisions but should not be the only basis for definitive therapy in complex cases.

The methodological revision made in this article is important for transparency. The aggregated estimates in Table 1 are descriptive; they are not pooled prevalence estimates from a formal meta‐analysis. This distinction matters because resistance surveillance studies are not directly comparable when they differ in population sampling, antifungal panels, breakpoint interpretation, and isolate phase. Future reviews should aim to build pathogen‐specific datasets that allow random‐effects meta‐analysis with confidence intervals and heterogeneity measures.

Policy responses should prioritize diagnostic access and antifungal stewardship. In many endemic regions, the main clinical problem is not only the existence of resistant fungi but also the inability to identify them early. Building reference laboratory networks, supporting AST capacity, using rapid antigen or molecular diagnostics where validated, and ensuring access to effective antifungals would reduce both mortality and unnecessary drug pressure. Stewardship should include human medicine, veterinary practice, and agricultural fungicide policy.

Finally, genomic surveillance should be scaled pragmatically. Whole‐genome sequencing is valuable for representative isolates and outbreak investigation, whereas targeted sequencing may be more feasible for routine detection of high‐confidence markers. Regional databases that link species identification, MIC values, resistance mutations, environmental source, and clinical outcome would improve the predictive value of genomic testing and support evidence‐based treatment guidelines in endemic settings.

## 5 Conclusion

Antifungal resistance in tropical mycoses is an emerging clinical and public health threat that requires coordinated surveillance, diagnostic improvement, and careful therapeutic stewardship. The strongest current evidence links azole resistance in *A. fumigatus* to environmental and agricultural selection, but clinically important nonsusceptibility is also reported in *H. capsulatum*, *Sporothrix* species, *T. marneffei*, and *Paracoccidioides* species. The revised analysis clarifies that numerical estimates are descriptive aggregations rather than outputs of formal meta‐analysis. Future work should standardize AST methods, develop validated breakpoints or epidemiological cutoff values for endemic dimorphic fungi, expand genomic resistance databases, and link laboratory data to patient outcomes.

## 6 Recommendations

1. Establish regional laboratory networks capable of species‐level identification and standardized AST for endemic fungi.
2. Use genomic assays as adjuncts to phenotypic testing, prioritizing high‐confidence resistance markers and representative whole‐genome sequencing.
3. Implement antifungal stewardship in hospitals and clinics, including dose optimization, adherence support, and therapeutic drug monitoring where feasible.
4. Develop One Health surveillance that samples clinical, veterinary, agricultural, and environmental reservoirs.
5. Create region‐specific treatment guidelines that account for drug availability, local resistance signals, and diagnostic capacity.
6. Build shared databases linking MICs, testing methods, molecular markers, geography, environmental exposure, and clinical outcome.

## Funding

The author has nothing to report.

## Ethics Statement

This review used published and publicly accessible data and did not require formal ethics approval.

## Conflicts of Interest

The author declares no conflicts of interest.

## Supporting information


**Supporting Table S1:** Data extraction template. This table reports the pre‐developed extraction fields used to collect information from the included studies. It is supplied to improve transparency and reproducibility.

## Data Availability

This review is based on published literature and publicly accessible data sources. Extracted summary data are available from the corresponding author upon reasonable request.
