# Supplementary material for: Emerging Antifungal Resistance in Tropical Mycoses: A Genomic and Clinical Perspective From Endemic Zones
Source: J R Soc N Z. 2026 Aug 10;56(4):e70074. doi: 10.1002/snz2.70074 (PMC13456969; doi:10.1002/snz2.70074)
Supplement: Supplementary file 1 [file SNZ2-56-e70074-s001.pdf]

Supplementary Table S1. Data extraction template

This table reports the pre-developed extraction fields used to collect information from included studies. It is supplied to improve transparency and reproducibility.

Domain	Data field	Description
Bibliographic information	Author, year, journal, DOI or accession	Identification of the source and linkage to reference manager record.
Study design	Clinical case series, surveillance, environmental sampling, genomic study, review	Classification of evidence type.
Setting	Country, region, endemic zone, hospital/environmental site	Used for spatial synthesis and hotspot mapping.
Pathogen	Species or species complex; identification method	Species-level naming where available; complex-level naming when not available.
Sample source	Clinical isolate, environmental isolate, animal-associated isolate, genomic repository	Used to separate clinical and One Health evidence.
Population / host	Patient group, immune status, HIV status, animal host, or environmental source	Used for clinical context and risk interpretation.
AST method	CLSI, EUCAST, YeastOne, E-test, modified microdilution, disk diffusion	Used to assess comparability of MIC or category data.
Antifungal agent	Drug tested and concentration range	Itraconazole, fluconazole, voriconazole, amphotericin B, echinocandins, or other agents.
MIC / category	MIC range, MIC50/MIC90, susceptible/resistant/non-wild-type category	Extracted as reported; categories not harmonized when breakpoints differed.
Genomic marker	ERG11, CYP51A, FKS1, ERG3, efflux genes, biofilm/stress markers	Used for genotype-phenotype correlation.
Clinical outcome	Failure, relapse, mortality, hospital stay, treatment change	Used only when explicitly reported.
Limitations	Breakpoint absence, duplicate isolates, small sample size, phase-dependent testing	Used in quality and bias interpretation.
